# In Silico Transcriptomic Analysis of Wound-Healing-Associated Genes in Malignant Pleural Mesothelioma

**DOI:** 10.3390/medicina55060267

**Published:** 2019-06-12

**Authors:** Erasmia Rouka, Eleftherios Beltsios, Dimos Goundaroulis, Georgios D. Vavougios, Evgeniy I. Solenov, Chrissi Hatzoglou, Konstantinos I. Gourgoulianis, Sotirios G. Zarogiannis

**Affiliations:** 1 Department of Transfusion Medicine, Faculty of Medicine, University of Thessaly, BIOPOLIS, 41500 Larissa, Greece; errouka@uth.gr; 2 Department of Physiology, Faculty of Medicine, University of Thessaly, BIOPOLIS, 41500 Larissa, Greece; elbeltsios@uth.gr (E.B.); chatz@med.uth.gr (C.H.); 3 Center for Integrative Genomics, University of Lausanne, 1015 Lausanne, Switzerland; Dimoklis.Gkountaroulis@unil.ch; 4 Swiss Institute of Bioinformatics, 1015 Lausanne, Switzerland; 5 Department of Neurology, Athens Naval Hospital, 11521 Athina, Greece; gvavou@uth.gr; 6 Institute of Cytology and Genetics of the Siberian Branch of the Russian Academy of Sciences, Novosibirsk 630090, Russia; 7 Novosibirsk State University, Novosibirsk 630090, Russia; eugsol@bionet.nsc.ru; 8 Department of Respiratory Medicine, Faculty of Medicine, University of Thessaly, BIOPOLIS, 41500 Larissa, Greece; kgourg@med.uth.gr

**Keywords:** in silico, malignant pleural mesothelioma, miRNA, transcriptomics, wound healing

## Abstract

*Background and objectives:* Malignant pleural mesothelioma (MPM) is a devastating malignancy with poor prognosis. Reliable biomarkers for MPM diagnosis, monitoring, and prognosis are needed. The aim of this study was to identify genes associated with wound healing processes whose expression could serve as a prognostic factor in MPM patients. *Materials and Methods:* We used data mining techniques and transcriptomic analysis so as to assess the differential transcriptional expression of wound-healing-associated genes in MPM. Moreover, we investigated the potential prognostic value as well as the functional enrichments of gene ontologies relative to microRNAs (miRNAs) of the significantly differentially expressed wound-healing-related genes in MPM. *Results:* Out of the 82 wound-healing-associated genes analyzed, 30 were found significantly deregulated in MPM. Kaplan–Meier analysis revealed that low *ITGAV* gene expression could serve as a prognostic factor favoring survival of MPM patients. Finally, gene ontology annotation enrichment analysis pointed to the members of the hsa-miR-143, hsa-miR-223, and the hsa-miR-29 miRNA family members as important regulators of the deregulated wound healing genes. *Conclusions:* 30 wound-healing-related genes were significantly deregulated in MPM, which are potential targets of hsa-miR-143, hsa-miR-223, and the hsa-miR-29 miRNA family members. Out of those genes, ITGAV gene expression was a prognostic factor of overall survival in MPM. Our results highlight the role of impaired tissue repair in MPM development and should be further validated experimentally.

## 1. Introduction

Malignant pleural mesothelioma (MPM) is a highly aggressive tumor of the pleural mesothelium, a metabolically active cell monolayer covering the lungs and the chest wall [1]. Neoplastic transformation of pleural mesothelial cells (PMCs) develops in the course of asbestos-induced chronic inflammation while genetic susceptibility factors, radiation exposure, and SV40 infection have also been indicated as co-factors in this process [2,3]. Recent evidence from animal studies and a few case series show that engineered nanoparticles, whose use is constantly expanding, can lead to a pathology similar to MPM and, thus, potentially lead to an increase of MPM incidence in the future and the same is the case with air pollution, which has been shown to lead to lung cancer [4,5].

Diagnosis of MPM is challenging mainly because the pleura is a common site for metastasis and reactive, benign changes observed in the pleural space may be confused with MPM [6]. At the same time, prognosis is poor since MPM therapeutic management depends on patient performance status, which is usually not good due to late diagnosis, and is potentially effective mostly in the epitheliod subtype [7]. For these reasons, the availability of effective bio markers is necessary for three clinical aspects of MPM: Early diagnosis, prognosis, and treatment outcome prediction [8].

Aberrant inflammation that could occur during wound healing has been linked to the pathogenesis of a variety of malignancies [9,10]. Environmental and infectious agents are considered key players during carcinogenesis development as they conduce to tissue damage and inflammatory reactions [9]. In the case of MPM, asbestos-induced chronic inflammation—mainly due to the production of reactive oxygen/nitrogen species—results in decreased tumor immunity [11]. Location and inflammatory response type impact on the overall prognosis of MPM patients [9].

PMCs hold a central role in the initiation and resolution of serosal inflammation and repair by secreting various pro- and anti- inflammatory mediators along with immunomodulatory ones [12,13]. It is worth noting that mesothelial regeneration of injured mesothelial surfaces appears diffusely across the traumatized area in contrast to epithelial-like surfaces, in which healing occurs solely from the wound edges [12]. Mesothelial injury and impaired healing can lead to the development of pleural effusions, serosal adhesions, and malignant mesothelioma [13,14]. Several studies have confirmed that following damage, mesothelial cells undergo mesothelial-to-mesenchymal transition (MMT), a process similar to epithelial-to-mesenchymal transition (EMT) [15]. It has been reported that the aggressiveness of MPM may be explained by its partial fibroblastic phenotype in the context of EMT conferring both high invasiveness and chemoresistance [16].

Although the above observations highlight the importance of normal serosal repair following injury, a thorough investigation of the exact role of wound-healing-associated genes in MPM development has not been performed [15]. Research in this direction is even more imperative nowadays due to the wide use of nanoparticles in a variety of applications since nano-sized fibrous particulates have similar properties to asbestos thus raising safety concerns for human health [17,18].

Here, we aimed at the identification of the differential expression of wound-healing-associated genes in MPM. For this purpose, we used established data mining techniques and transcriptomic analysis [19,20,21,22]. Moreover, we investigated their potential prognostic value as well as the functional enrichments of gene ontologies (GO) relative to the microRNAs (miRNAs) that regulate the significantly differentially expressed wound-healing-related genes in MPM.

## 2. Materials and Methods

### 2.1. Transcriptomic Analysis of Wound-Healing-Associated Genes in MPM

The Oncomine Cancer Microarray database Premium Research Edition (http://www.oncomine.org) was used to investigate the expression profile of wound-healing-associated genes in MPM compared with healthy controls. Gene expression data from the GDS1220 dataset of GEO Profiles were used in which the Affymetrix Human Genome U133A array was used assessing 12,624 genes [23]. We analyzed available data for 82 wound-healing-related genes (from the according list of genes included in the Wound Healing RT^2^ Profiler PCR Arrays from Qiagen) that were assessed in the GDS1220 study (Table 1), so as to detect gene expression differences between MPM specimens and healthy controls [23]. The raw data were downloaded in Excel format from Oncomine and only the ones referring to surgically excised samples were selected, leading to the use of *n* = 40 MPM cases and *n* = 9 controls (*n* = 5 pleura and *n* = 4 lung samples). The gene expression data were log transformed, median centered per array, and the standard deviation was normalized to one per array as described previously [24].

### 2.2. Evaluation of the Significantly Differentially Expressed Wound-Healing-Related Genes for Prognostic Relevance

We investigated the prognostic relevance of the significantly differentially expressed wound-healing-related genes by creating survival curves, using the publicly available survival data of each patient from the GDS1220 study. Patients were grouped as high expressing (above median) and low expressing (below median) per each significantly differentially expressed gene based on the median value of gene expression. To further evaluate the prognostic significance of the genes that were identified in the analysis of the GDS1220 mesothelioma study, we constructed survival curves from data derived from The Cancer Genome Atlas (TCGA). For this analysis, we used the PROGgeneV2 Prognostic Database software following the same approach used previously [25].

### 2.3. Functional Annotation Enrichment Gene Ontology Analysis of the miRNAs That Regulate the Significantly Differentially Expressed Wound-Healing-Related Genes in MPM

Functional annotation enrichment analysis of GO relative to Biological Functions and miRNAs was performed, assuming the statistical background of the whole genome. The complete list of the significantly differentially expressed wound-healing-related genes was introduced to the portal ToppFun, an application of the ToppGene Suite (https://toppgene.cchmc.org/). ToppFun reports functional enrichment of input gene lists based on transcriptome (gene expression), proteome (protein domains and interactions), regulome (transcription factor binding sites and miRNA), ontologies (GO, pathway), phenotype (human disease and mouse phenotype), pharmacome (drug–gene associations), and bibliome (literature co-citation) [26]. Functional enrichments are provided by the ToppFun algorithm, which employs hypergeometric distribution with multiple correction testing (false discovery rate; FDR) to determine statistical significance. Analysis was performed during February 2019.

### 2.4. Statistical Analysis

The results were analyzed using GraphPad Prism 8.0 (GraphPad Software, San Diego, CA) and R 3.3.2 [27]. Data distribution was tested by the Kolmogorov–Smirnov normality test. Comparisons of gene expressions were performed with the one-tailed t-test for parametric data and the Mann–Whitney U-test for nonparametric data. The Benjamini–Hochberg FDR was employed for multiple correction testing, which reports FDR (or q-value), in order to corroborate the validity of the results. The calculation of the q statistic was based on the formula Q = P value × total number of genes/P-value rank [24]. Statistical significance was set at the *q* < 0.05 level. Kaplan–Meier survival curves were generated using the overall survival data of the MPM patients and by grouping them into high and low gene expressions based on the median. The log-rank (Mantel–Cox) test, which gives equal weight to deaths at all time points, was used. Differences were deemed significant with a *p* ≤ 0.05. A multivariate regression was performed using the initial dataset of 30 differentially expressed genes where the ITGAV gene expression was chosen as the dependent variable. At first, all predictors were considered, and a model was derived. This mode had an acceptable R2 value but had to be discarded since the predictors were over-fit. Next, a backwards method was implemented that takes into consideration the Variance Inflation Factor (VIF) value of each predictor. If VIF of an independent variable exceeded the predetermined threshold value of 5, that predictor was discarded from the model. Thus, one avoids multi-collinearity in a model but may still obtain an overfitted model. To avoid this, an additional backwards step-wise regression was performed on the surviving variable so as to have a robust final model.

## 3. Results

### 3.1. Identification of the Differential Transcriptional Expression Of Wound-Healing-Associated Genes in MPM

Out of the 82 wound-healing-related genes analyzed, 30 were found significantly differentially expressed in MPM (14 up-regulated/16 down-regulated, results summarized in Table 2). Ten genes (ITGB3, ITGB5, COL5A3, VEGFA, WNT5A, WISP1, CTNNB1, F13A1, PLAUR, TIMP1) were found significantly deregulated in the initial mean value comparison test (*p* = 0.008, *p* = 0.023, *p* = 0.016, *p* = 0.0115, *p* = 0.0205, *p* = 0.0055, *p* = 0.024, *p* = 0.0445, *p* = 0.0405, *p* = 0.046, respectively) but not after the multiple correction testing (*q* = 0.055, *q* = 0.1649, *q* = 0,128, *q* = 0.1905, *q* = 0.314, *q* = 0.055, *q* = 0.133, *q* = 0.1898, *q* = 0.1789, *q* = 0.1375, respectively).

### 3.2. Prognostic Significance of ITGAV Gene Expression in MPM

**Figure 1:**
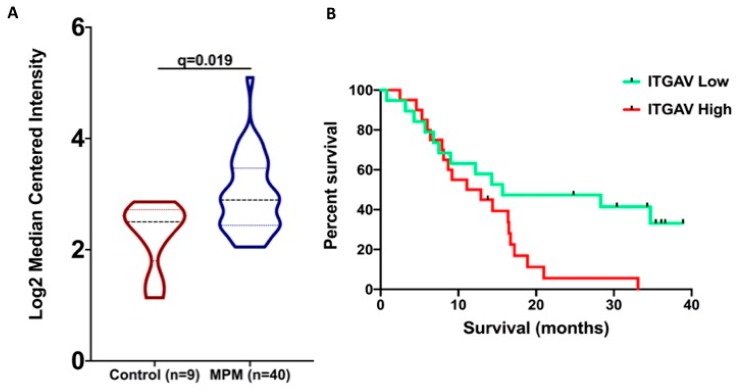
(**A**) Violin plot of the *ITGAV* gene expression shows that it is significantly overexpressed in MPM patients compared to controls. (**B**) Low ITGAV gene expression in MPM patients favors their survival (shown in months).

**Figure 2:**
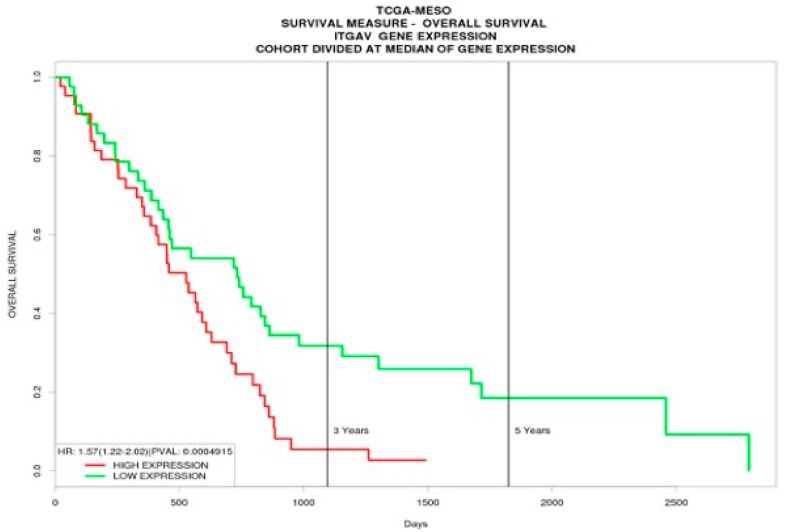
Results of Kaplan–Meier survival analysis from The Cancer Genome Atlas (TCGA) mesothelioma dataset derived from PROGgeneV2 database, shows that low ITGAV gene expression favors survival (shown in days) in MPM patients.

Kaplan–Meier analysis of the significantly deregulated wound-healing-related genes in MPM identified the ITGAV gene expression as a predictor of overall survival. Graphical representation of the ITGAV gene expression from the GDS1220 study is shown in Figure 1A. In the GDS1220 study, MPM patients with low ITGAV expression had a median overall survival of 15.7 months versus 12 months of those that had high expression (*p* = 0.0263) (Figure 1B). Analysis from the TCGA patient data derived from the PROGgeneV2 database corroborates that low ITGAV gene expression favors survival in MPM patients (*p* = 0.001) (Figure 2).

### 3.3. Statistical Modeling Reveals A Positive Correlation of ITGAV and COL5A1 Gene Expressions

Multivariate regression was performed as shown in Table 3, and the following model was obtained using backwards selection methods:

*ITGAV* = 1.89725 + 0.41537 *COL5A1* (1)

The model explains the 43.54% (adjusted R^2^ = 0.4354) of the variability of the response data around its mean. F-statistic and *p*-value indicate that the model is significant for the explanation of the variability of the response data. The coefficients of the model are positive, hence whenever the value of COL5A1 increases, the same happens to the value of ITGAV. The value of each coefficient shows the increase of the value of ITGAV if the corresponding variable is increased or decreased by one unit, while the rest remain fixed.

### 3.4. Enriched Gene Ontologies (GO) Relative To Regulating miRNAs of the Significantly Differentially Expressed Wound-Healing-Associated Genes In MPM

The complete set of the significantly differentially expressed wound-healing-associated genes was entered in the ToppFun software. The top five enriched GO for biological functions are presented in Table 4, and the miRNAs that regulate the differentially expressed genes are shown in Table 5.

## 4. Discussion

Chronic tissue repair and insistent immune system stimulation following the accumulation of inhaled asbestos fibers in the pleural space are key oncogenic processes during MPM development [28]. In this study, we used transcriptome data mining in order to assess the differential mRNA expression of wound-healing-related genes in MPM. We analyzed available data for 82 genes, of which 30 were found significantly deregulated in MPM specimens compared with healthy tissues. Identified genes are mainly involved in inflammation (CXCL2, CXCL5, IL6, IL10), cellular adhesion (ITGA3, ITGAV, ITGB6), tissue remodeling (CTSG, F3, PLG, SERPINE1), and extracellular matrix organization (COL1A1, COL3A1, COL5A1, COL5A2, CSF3, HBEGF, MIF, TGFA, VTN), fundamental processes in the induction of EMT and cancer progression [29,30,31,32]. A recent study has suggested that asbestos induces mesothelial-to-fibroblastic transition in an inflammasome-dependent manner and this seems to apply for other pathogenic particles as well [28,33,34].

The acquisition of a mesenchymal phenotype by PMCs has been correlated with the sarcomatoid histological type of MPM, the latter being associated to worse prognosis [16,35]. It has been suggested that in MPM patients with sarcomatoid histology, radical surgery should be excluded, and therapy should aim at symptom control and preservation of quality of life [36]. Considering the importance of clear histological subtype distinction in the initiation of the appropriate therapeutic intervention, it would be of great interest to investigate the transcriptional expression of the 30 significantly deregulated genes—identified in this study—with respect to the MPM phenotype [22]. Unfortunately, data relative to the histological subtype of MPM specimens were not available in the GDS1220 mesothelioma study.

Among the 30 significantly differentially expressed wound-healing-associated genes in MPM, ITGAV was identified as a predictor of overall survival in patients suffering from this type of cancer. In the transcriptomic analysis, ITGAV was found significantly up-regulated in MPM subjects compared with healthy controls, and survival analysis revealed that low ITGAV gene expression favors the survival of MPM patients. In accordance with our results, recent studies have reported the tumor-promoting effect of ITGAV via the furtherance of the EMT process [37,38]. The gene expression level of the ITGAV integrin has also been associated with global survival in patients with colorectal cancer, both in univariate and multivariate analyses [39].

Following the above findings, our regression modelling predicted a significant positive correlation in the expression profiles of the ITGAV and COL5A1 genes. The latter encodes the a1 chain of type V collagen, a fibril-forming collagen that is mainly distributed in the lung, cornea, bone, and fetal membranes, together with type I collagen [40,41]. Interestingly, COL5A1 has been included in a 10-gene signature that is associated with poor survival both in patients with high-grade serous ovarian cancer and renal cell carcinoma [42,43].

Finally, the biological functions mediated by the differentially regulated genes as revealed by the GO functional enrichment analysis were pertinent to wound healing functions, such as cell locomotion, mobility, and migration. Furthermore, the GO annotation enrichment analysis predicted that the 30 wound-healing-related genes that were found significantly deregulated in MPM might be targets of the hsa-miR-143, hsa-miR-223, and the hsa-miR-29 miRNA family members. MicroRNAs have emerged as significant players in the biology of MPM, whilst miRNA-based therapy for the treatment of MPM has been suggested as an exciting area of research [44]. The hsa-miR-29c has been identified as an epigenetic regulator in MPM via the down-regulation of DNA methyltransferases and the up-regulation of demethylating genes [45]. In addition, increased expression of this miRNA has been linked to a more favorable prognosis in MPM [45]. On the other hand, hsa-miR-143 has been linked to MPM only once, in a study that showed that its expression is significantly reduced in MPM as compared to non-neoplastic corresponding tissue [46]. Its functional role in MPM is unknown, however in other cancers, the decreased expression of has-miR-143 has been shown to promote EMT, tumor growth, and metastasis [47,48]. Overall, these reports strengthen the findings of our in silico transcriptomic analysis implicating 30 wound-healing-associated genes in MPM pathogenicity. However, it is imperative to investigate the transcriptional expression of these genes in the clinical setting, taking into account both MPM phenotype and history of asbestos exposure.

## 5. Conclusions

Conclusively, using data mining and transcriptomic analysis, we have identified a significant deregulation of 30 wound-healing-related genes in MPM specimens compared to healthy tissues. We have also demonstrated that these genes are possible targets of the hsa-miR-143, hsa-miR-223, and the hsa-miR-29 miRNA family members, which contribute in MPM pathobiology as evidenced by recent studies. Finally, we have identified ITGAV as a novel predictor of overall survival in MPM. Our results highlight the role of impaired tissue repair in MPM development and should be further validated experimentally.

## Figures and Tables

**Table 1:** The wound-healing-associated genes investigated in this study.

Hugo Gene Nomenclature Committee Gene Name.	Gene Description
**ITGA3**	Integrin Subunit Alpha 3
**ITGAV**	Integrin Subunit Alpha V
**ITGB6**	Integrin Subunit Beta 6
**RAC1**	Ras-Related C3 Botulinum Toxin Substrate 1 (Rho Family, Small GTP Binding Protein Rac1)
**COL5A1**	Collagen Type V Alpha 1 Chain
**COL5A2**	Collagen Type V Alpha 2 Chain
**ANGPT1**	Angiopoietin 1
**COL1A1**	Collagen Type I Alpha 1 Chain
**COL3A1**	Collagen Type III Alpha 1 Chain
**CSF3**	Colony Stimulating Factor 3
**HBEGF**	Heparin Binding EGF Like Growth Factor
**MIF**	Macrophage Migration Inhibitory Factor (Glycosylation-Inhibiting Factor)
**TGFA**	Transforming Growth Factor Alpha
**TNF**	Tumor Necrosis Factor
**VTN**	Vitronectin
**CXCL2**	C-X-C Motif Chemokine Ligand 2
**CXCL5**	C-X-C Motif Chemokine Ligand 5
**IL6**	Interleukin 6
**IL10**	Interleukin 10
**PTGS2**	Prostaglandin-Endoperoxide Synthase 2
**MAPK3**	Mitogen-Activated Protein Kinase 3
**PTEN**	Phosphatase And Tensin Homolog
**IL6ST**	Interleukin 6 Signal Transducer
**STAT3**	Signal Transducer And Activator Of Transcription 3
**TGFBR3**	Transforming Growth Factor Beta Receptor 3
**CTSG**	Cathepsin G
**F3**	Coagulation Factor III, Tissue Factor
**F13A1**	Coagulation Factor XIII A Chain
**PLAUR**	Plasminogen Activator, Urokinase Receptor
**PLG**	Plasminogen
**SERPINE1**	Serpin Family E Member 1
**TIMP1**	IMP Metallopeptidase Inhibitor 1
**COL1A2**	Collagen Type I Alpha 2 Chain
**ITGB3**	Integrin Subunit Beta 3
**ITGB5**	Integrin Subunit Beta 5
**COL5A3**	Collagen Type V Alpha 3 Chain
**VEGFA**	Vascular Endothelial Growth Factor A
**WNT5A**	Wnt Family Member 5A
**WISP1**	WNT1 Inducible Signaling Pathway Protein 1
**CTNNB1**	Catenin Beta 1
**MAPK1**	Mitogen-Activated Protein Kinase 1
**EGFR**	Epidermal Growth Factor Receptor
**TGFB1**	Transforming Growth Factor Beta 1
**CDH1**	Cadherin 1
**ITGA1**	Integrin Subunit Alpha 1
**ITGA2**	Integrin Subunit Alpha 2
**ITGA4**	Integrin Subunit Alpha 4
**ITGA5**	Integrin Subunit Alpha 5
**ITGA6**	Integrin Subunit Alpha 6
**ITGB1**	Integrin Subunit Beta 1
**ACTA2**	Actin, Alpha 2, Smooth Muscle, Aorta
**ACTA1**	Actin, Alpha 1, Skeletal Muscle
**RHOA**	Ras Homolog Family Member A
**TAGLN**	Transgelin
**COL4A1**	Collagen Type IV Alpha 1 Chain
**COL4A3**	Collagen Type IV Alpha 3 Chain
**COL14A1**	Collagen Type XIV Alpha 1 Chain
**CTSK**	Cathepsin K
**CTSL2**	cathepsin L2
**FGA**	Fibrinogen Alpha Chain
**MMP1**	Matrix Metallopeptidase 1
**MMP2**	Matrix Metallopeptidase 2
**MMP7**	Matrix Metallopeptidase 7
**MMP9**	Matrix Metallopeptidase 9
**PLAT**	Plasminogen Activator, Tissue Type
**PLAU**	Plasminogen Activator, Urokinase
**CSF2**	Colony Stimulating Factor 2
**CTGF**	Connective Tissue Growth Factor
**EGF**	Epidermal Growth Factor
**FGF2**	Fibroblast Growth Factor 2
**FGF7**	Fibroblast Growth Factor 7
**HGF**	Hepatocyte Growth Factor
**IGF1**	Insulin Like Growth Factor 1
**PDGFA**	Platelet Derived Growth Factor Subunit A
**CCL2**	C-C Motif Chemokine Ligand 2
**CCL7**	C-C Motif Chemokine Ligand 7
**CD40LG**	CD40 Ligand
**CXCL1**	C-X-C Motif Chemokine Ligand 1
**CXCL11**	C-X-C Motif Chemokine Ligand 11
**IL1B**	Interleukin 1 Beta
**IL2**	Interleukin 2
**IL4**	Interleukin 4

**Table 2:** Wound-healing-associated genes differentially expressed in malignant pleural mesothelioma (MPM) patients.

	Q Value	FC
**Up-Regulated**		
***ITGA3***	*0.0075*	*3.65983*
***ITGAV***	*0.019*	*1.62136*
***RAC1***	*0.0075*	*1.437*
***COL5A1***	*1.29 × 10^−3^*	*3.4584*
***COL5A2***	*1.06 × 10^−3^*	*3.7013*
***COL1A1***	*1.24 × 10^−3^*	*2.6257*
***COL3A1***	*2.79 × 10^−3^*	*4.0285*
***MIF***	*8.36 × 10^−3^*	*2.8341*
***VTN***	*4.55 × 10^−3^*	*7.1654*
***MAPK3***	*1.24 × 10^−3^*	*2.4605*
***IL6ST***	*0.0149*	*1.6038*
***STAT3***	*2.51 × 10^−3^*	*2.9051*
***SERPINE1***	*0.02*	*3.1149*
***COL1A2***	*0.0255*	*2.7937*
**Down-Regulated**		
***ITGB6***	*9.76 × 10^−4^*	*−2.5496*
***ANGPT1***	*0.011*	*−2.3463*
***CSF3***	*0.012*	*−3.5254*
***HBEGF***	*0.02*	*−1.5826*
***TGFA***	*0.0126*	*−1.4865*
***TNF***	*0.04208*	*−4.0594*
***CXCL2***	*3.86 × 10^−3^*	*−4.6691*
***CXCL5***	*8.40 × 10^−4^*	*−2.1452*
***IL6***	*0.01932*	*−6.2047*
***IL10***	*0.039*	*−1.3583*
***PTGS2***	*7.29 × 10^−4^*	*−4.408*
***PTEN***	*0.033*	*−1.407*
***TGFBR3***	*2.14 × 10^−3^*	*−2.0823*
***CTSG***	*2.08 × 10^−3^*	*−2.5715*
***F3***	*0.03*	*−2.3331*
***PLG***	*0.027*	*−1.5597*

**Table 3:** Statistical modeling of the correlation of ITGAV and COL5A1 gene expressions.

Coefficients	Estimate	Std. Error	T Value	Pr (>|t|)
(Intercept)	1.89725	0.20885	9.084	4.59 × 10^−11^ ***
COL5A1	0.41537	0.07451	5.574	2.18 × 10^−6^ ***

**Table 4:** Functional enrichment analysis relative to Biological Functions of the wound-healing-associated genes differentially expressed in MPM patients *.

	ID	Name	P-Value	FDR B&H	FDR B&Y	Bonferroni	Genes From Input	Genes in Annotation
1	GO:0040011	locomotion	2.531 × 10^−23^	7.177 × 10^−20^	6.120 × 10^−19^	7.177 × 10^−20^	26	1735
2	GO:0016477	cell migration	5.798 × 10^−23^	1.644 × 10^−19^	24	1300		
3	GO:0051674	localization of cell	5.395 × 10^−22^	3.825 × 10^−19^	3.262 × 10^−18^	1.530 × 10^−18^	24	1428
4	GO:0048870	cell motility	5.395 × 10^−22^	3.825 × 10^−19^	3.262 × 10^−18^	1.530 × 10^−18^	24	1428
5	GO:0030334	regulation of cell migration	1.635 × 10^−21^	9.274 × 10^−19^	7.909 × 10^−18^	4.637 × 10^−18^	20	742

* Results were obtained through ToppFun, an application of the ToppGene suite (https://toppgene.cchmc.org/). FDR: False discovery rate; B&H: Benjamini–Hochberg; B&Y: Benjamini–Yekutieli. Only the top five results are presented.

**Table 5:** Functional enrichment analysis relative to miRNAs of the wound-healing-associated genes differentially expressed in MPM patients *.

	ID	Name	Source	P-Value	FDR B&H	FDR B&Y	Bonferroni	Genes From Input	Genes in Annotation
1	hsa-miR-143-3p:Functional MTI	Functional MTI	miRTarbase	5.442 × 10^−12^	1.459 × 10^−8^	1.236 × 10^−7^	1.459 × 10^−8^	7	228
2	hsa-miR-223-3p:Functional MTI	Functional MTI	miRTarbase	5.747 × 10^−10^	7.704 × 10^−7^	6.526 × 10^−6^	1.541 × 10^−6^	5	98
3	hsa-miR-29b-3p:Functional MTI	Functional MTI	miRTarbase	1.159 × 10^−9^	1.036 × 10^−6^	8.776 × 10^−6^	3.108 × 10^−6^	6	261
4	hsa-miR-29a:PITA	hsa-miR-29a:PITA TOP	PITA	3.728 × 10^−9^	1.666 × 10^−6^	1.411 × 10^−5^	9.995 × 10^−6^	7	583
5	hsa-miR-29c:PITA	hsa-miR-29c:PITA TOP	PITA	3.728 × 10^−9^	1.666 × 10^−6^	1.411 × 10^−5^	9.995 × 10^−6^	7	583

* Results were obtained through ToppFun, an application of the ToppGene suite (https://toppgene.cchmc.org/). FDR: False discovery rate; B&H: Benjamini–Hochberg; B&Y: Benjamini–Yekutieli. Only the top five results are presented.
